# The impact of pathologic differentiation (well/poorly) and the degree of Ki-67 index in patients with metastatic WHO grade 3 GEP-NECs

**DOI:** 10.18632/oncotarget.18168

**Published:** 2017-05-25

**Authors:** Hee Kyung Kim, Sang Yun Ha, Jeeyun Lee, Se Hoon Park, Joon Oh Park, Ho Yeong Lim, Won Ki Kang, Young Suk Park, Seung Tae Kim

**Affiliations:** ^1^ Division of Hematology-Oncology, Department of Medicine, Samsung Medical Center, Sungkyunkwan University School of Medicine, Seoul, Korea; ^2^ Department of Pathology and Translational Genomics, Samsung Medical Center, Sungkyunkwan University School of Medicine, Seoul, Korea

**Keywords:** pathologic differentiation, Ki67 index, gastroenteropancreatic neuroendocrine carcinoma (GEP-NEC)s

## Abstract

We investigated the impact of pathologic differentiation (well or poorly differentiated) in metastatic grade 3 GEP-NEC patients receiving etoposide and platinum (EP)-based therapy, and evaluated a more exact Ki67 index cut-off point to select patients with grade 3 GEP-NEC who might benefit from EP-based therapy. A total of 31 patients with metastatic grade 3 GEP-NECs receiving EP-based therapy were included in this study. Primary sites included 13 foregut-derived GEP-NECs [stomach (*n =* 4), duodenum (*n =* 4), and pancreas (*n =* 5)] and 2 hindgut-derived GEP-NECs of the rectum. 14 patients had well differentiated (WD) and 17 had poorly differentiated (PD). Between WD and PD grade 3 GEP-NECs, there was a significant difference in the distribution of Ki67 index. There was no significant difference of treatment efficacy between WD and PD grade 3 GEP-NECs (RR; 35.7% vs. 41.2%, *p* = 0.525). Tumor response to EP occurred in 5 of 7 patients with Ki67 > 60% and 7 of 24 with Ki67 ≤ 60%, which was significantly different (RR; 71.4% vs. 29.2%, *P* = 0.043). Among grade 3 GEP-NECs, there was a significant difference in ranges of Ki67 index between WD and PD NECs. Higher levels (> 60%) of Ki67 index might be a predictive marker for efficacy of EP as a standard regimen in grade 3 GEP-NECs.

## INTRODUCTION

Neuroendocrine tumors (NETs) consist of a heterogeneous group of malignancies that arise from neuroendocrine system. The origin of NETs are mostly gastroenteropancreatic (GEP) which is arising in the foregut, midgut, or hindgut [1]. Even though NETs are rare malignancy, a rise in incidence of NETs is reported according to recent studies based on the Surveillance, Epidemiology, and End Results (SEER) cancer registry and European studies [2]. Clinical features of GEP-NET are very heterogeneous and nonspecific. GEP-NET is characterized by tumor grade or differentiation, either indolent or aggressive [2, 3]. According to the World Health Organization (WHO) classification, NETs are generally classified into 2 subgroups, tumor (NET) and carcinoma (NEC). NET or NEC is determined by Ki67 index and/or mitotic index determined, < 20% Ki67 and/or < 20/high power field (HPF) mitotic count is classified as NET. Furthermore, NET is subdivided into Grade 1 (G1) or Grade 2 (G2) NET, tumors with Ki67 ≤ 2% and/or mitosis < 2/HPF are classified as G1 and those with Ki67 3–20% and/or mitosis 2–20/HPF are G2 [4]. NETs with Ki67 > 20% and/or mitosis > 20/HPF are classified into Grade 3 tumors (NECs), which comprise small-cell or large-cell carcinomas. Hence, grade 3 GEP-NECs show more proliferative and aggressive clinicopathologic features and have been regarded as synonymous with poorly differentiated neuroendocrine carcinoma, which encompass small-cell and large-cell subtypes. Currently, grade 3 metastatic GEP-NEC needs to be healed with cytotoxic agents. The standard treatment is platinum-based therapies combined with etoposide [5–7].

Recent studies have described the existence of uncommon well differentiated GEP-NETs that exhibit characteristic morphologic features of low or intermediate grade neoplasms but have a proliferative rate that breaches the threshold for WHO classification of G3 neuroendocrine neoplasms. Well differentiated and poorly differentiated groups of NETs have different clinical courses and different treatment outcomes, [8, 9] but less is known about treatment outcomes with etoposide plus platinum between well differentiated and poorly differentiated metastatic grade 3 GEP-NEC. In addition, a recent landmark study showed that grade 3 NECs with a Ki67 index less than 55% do not respond to platinum based chemotherapy, in contrast to grade 3 NEC with a Ki67 index greater than 55% [9].

Herein, we investigated the impact of pathologic differentiation (well or poorly differentiated) in metastatic grade 3 GEP-NEC patients receiving etoposide and platinum-based therapy. Simultaneously, we evaluated a more exact Ki67 index cut-off point to select patients with grade 3 GEP-NEC who might benefit from etoposide plus platinum-based therapy

## RESULTS

### Patient characteristics

Among patients who were diagnosed with metastatic grade 3 GEP-NECs at Samsung Medical Center between June 2013 and March 2016, 31 GEP-NEC patients receiving etoposide and platinum-based therapy were analyzed in this study. Table 1 showed baseline characteristics of these 31 patients. The median age of the patients was 58.0 years (range, 26–86) and the majority of patients was male (male to female ratio; 2.44). Primary sites included 13 foregut-derived GEP-NECs [stomach (*n* = 4), duodenum (*n* = 4), and pancreas (*n* = 5)], and 2 hindgut-derived GEP-NECs of the rectum. Sixteen unclassified GEP-NETs originated from 7 gall-bladder (GB), 6 liver and 3 unknown primary sites. According to pathologic differentiation, 14 patients had well differentiated and 17 had poorly differentiated grade 3 GEP-NECs (Supplementary Table 1). Between well differentiated and poorly differentiated grade 3 GEP-NECs, there was a significant difference for the distribution of Ki67 index (Table 2).

**Table 1:** Clinicopathologic features of grade 3 metastatic gastroenteropancreatic (GEP)-neuroendocrine neoplasm patients (N = 31)

**Variables**		**No**	**%**
**Gender**	Male	22	70.9
	Female	9	29.1
**Age**	Median (Range)	58.0 (26–86)	
**Differentiation**	Well	14	45.2
	Poorly	17	54.8
**Primary Site**	Foregut	13	41.9
	Midgut	-	0.0
	Hindgut	2	6.5
	Unclassified	16	51.6
**Primary Organ**	Stomach	4	12.9
	Duodenum	4	12.9
	Pancreas	5	16.1
	Gall bladder	7	22.6
	Liver	6	19.4
	Rectum	2	6.5
	Unknown primary site	3	9.7
**No. of metastatic sites**	1	16	51.6
	1 <	15	48.4
**Metastatic site**	Liver	22	70.9
	Lymph node	14	45.2
	Lung	6	19.4
	Ovary	2	6.5

**Table 2:** Distribution of Ki67 (%) between well differentiated and poorly differentiated grade 3 GEP-neuroendocrine neoplasms

**Ki67**	**Well Differentiated**	**Poor Differentiated**	***P*-value**
**Range**	28%−60%	30%−85%	0.000
**Median**	30%	60%	

### Treatment efficacy

As first-line chemotherapy, 31 patients with grade 3 metastatic GEP-NEC received etoposide plus cisplatin. Complete response (CR) was achieved in one patient (3.2%). Eleven partial responses (35.5%) were observed, for a response rate (RR) of 38.7%. Table 3 shows the comparison of treatment efficacy of etoposide plus cisplatin in 31 patients according to pathologic differentiation (well differentiated vs. poorly differentiated). There was no significant difference of treatment efficacy between well and poorly differentiated grade 3 GEP-NECs (RR; 35.7% vs. 41.2%, *p* = 0.525). Table 4 shows the difference of treatment response according to Ki67 index (Ki67 > 60% vs. Ki67 ≤ 60%). Tumor response to etoposide plus cisplatin occurred in 5 of 7 patients with Ki67 > 60% and 7 of 24 with Ki67 ≤ 60%, which was significantly different (RR; 71.4% vs. 29.2%, *P* = 0.043).

**Table 3:** Tumor response to etoposide plus cisplatin in 31 grade 3 metastatic GEP-neuroendocrine neoplasm patients according to pathologic differentiation

		Well Differentiated	Poor Differentiated	*P*-value
**Etoposide plus Cisplatin as first line treatment**	**Complete Response (CR)**	0	1	
	**Partial Response (PR)**	5	6	
	**Stable Disease (SD)**	7	4	
	**Progressive Disease (PD)**	2	6	
	**Response Rate (CR+PR)/31 (%)**	35.7%	41.2%	0.525

**Table 4:** Tumor response to etoposide plus cisplatin in patients according to Ki67 index

		Ki67 > 60%	Ki67 ≤ 60%	*P*-value
**Etoposide plus Cisplatin**	Response (CR/PR)	5	7	0.043
	Non-response (SD/PD)	2	17	
	Total (*N* = 31)	7	24	

### The impact of pathologic differentiation and Ki67 index on progression-free survival

**Figure 1:**
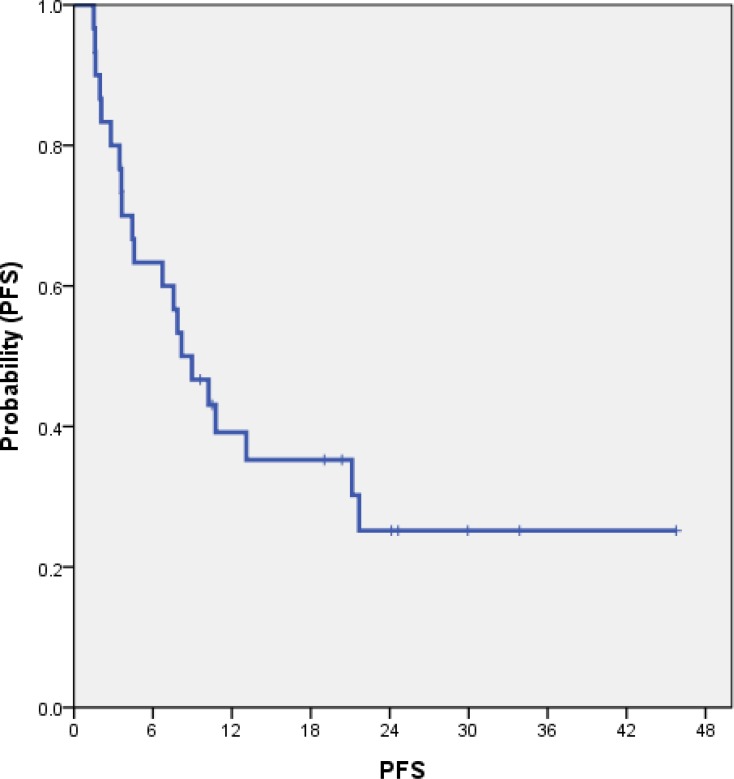

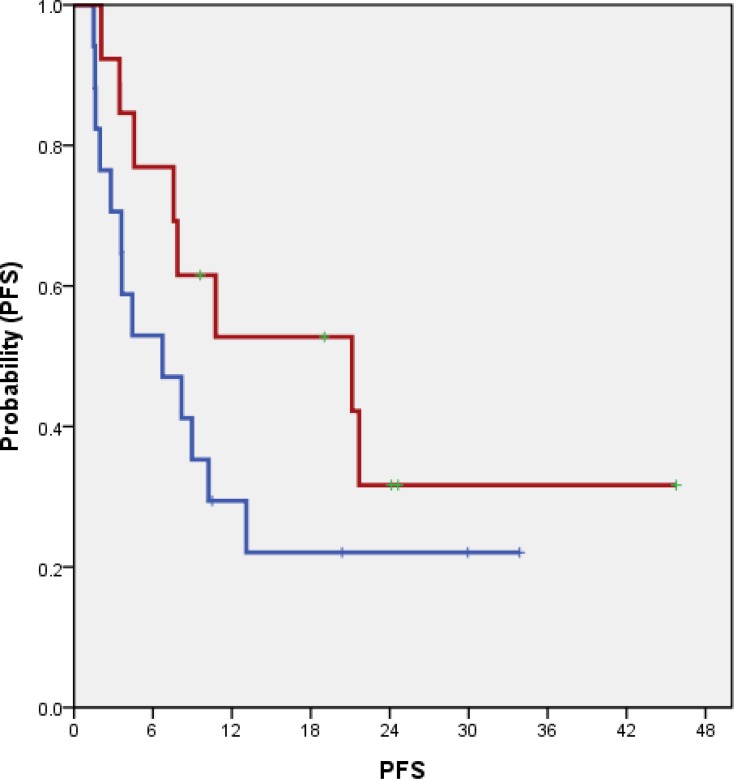

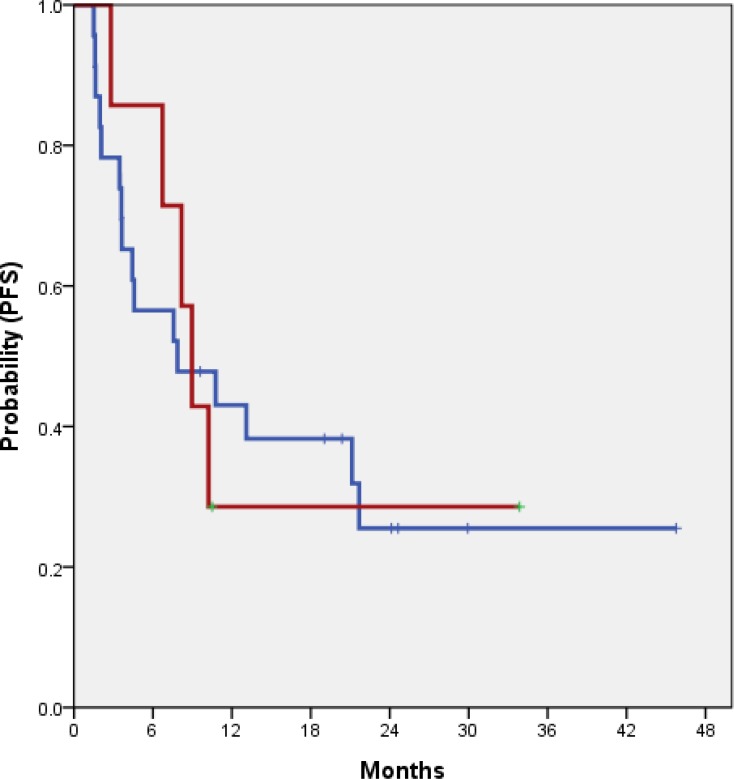
Progression free survival in 31 grade 3 GEP-NETs **(A)** and according to pathologic differentiation (B). (A) PFS: 8.2 months (95% CI, 4.7–11.7). **(B)** *P* = 0.163, W.D. PFS: 21.2 months (95% CI, 1.3–40.9), P.D. PFS: 6.7 months (95% CI, 0.7–12.8). (C) *P* = 0.959, Ki67 ≤ 60% PFS: 7.87 months (95% CI, 0.0–17.26), Ki67 > 60%. PFS: 8.97 months (95% CI, 6.91–11.02).

In 31 patients receiving etoposide plus cisplatin, the median progression-free survival (PFS) was 8.2 months (95% CI 4.7–11.7) (Figure 1A). There was no statistically a difference in PFS according to pathologic differentiation (well differentiated vs. poorly differentiated; median PFS; 21.2 vs. 6.7 months, respectively, *P* = 0.163) (Figure 1B). In addition, there was a statistical difference in PFS between patients with Ki67 index > 60% and ≤ 60% (median PFS; 7.87 vs. 8.97 months, respectively, *P* = 0.959) (Figure 1C).

## DISCUSSION

Cytotoxic chemotherapy is the main tool for metastatic grade 3 GEP-NEC. Etoposide plus platinum (EP) has been considered as standard regimen in NEC, based on similarities between NEC and small cell lung cancer (SCLC) [10]. However, previous studies report an RR of first line chemotherapy of 14−67% for EP [11–14]. These diverse outcomes reflect the heterogeneity of GEP-NECs. The tumor grade and/or differentiation determine characteristics of indolent or aggressive GEP-NET [2, 3]. Grade 3 GEP-NECs also include varied pathologic differentiation and a range of Ki67 indexes, but the influence of these heterogeneities on tumor response to EP is still unknown. Many studies analyzed Grade 3 category heterogeneity [3, 15–17]. The broad interval of grade 3 disease may include largely different neoplasms, with different responses to therapy. Currently, heterogeneity of 2010 WHO G3 GEP NECs is clearly a reality. The stratification of this category is still debated, but it is now proposed a combined factor (morphology and Ki67) to stratify G3 GEP NECs into three different categories, NEC type A, Type B and Type C in terms of survival.

We observed that the EP regimen yielded a superior tumor response in Ki67 > 60% grade 3 GEP-NECs than in those with Ki67 ≤ 60%. This finding was consistent with a recent landmark study [9]. In this analysis, all GEP-NECs with Ki67 > 60% were poorly differentiated. Of course, morphologically well differentiated GEP-NETs have a proliferative rate (Ki67 index) that meets the threshold for grade 3 GEP-NECs. However, well differentiated GEP-NETs are regarded as tumors with the Ki67 of lower range. In the present analysis, the median value of Ki67 index among well differentiated tumors was 20%, compared to 60% for poorly differentiated tumors. We also analyzed the tumor response to EP according to pathological differentiation. Among grade 3 GEP-NECs, pathologic differentiation did not affect tumor response to EP. Thus, these findings suggest that tumor response to EP might be caused by proliferative rate, but not pathologic differentiation, in grade 3 GEP-NECs.

There are many differences in both clinicopathological and genomic aspects of well and poorly differentiated NETs [18]. Genomic analysis have discovered the recurrent and mutually exclusive DAXX and ATRX mutations in approximately 44% of well differentiated pancreatic NETs [18–20]. These genomic characteristics are specific for well differentiated NETs and have not been seen in poorly differentiated NETs. In contrast, poorly differentiated NECs have genotypic alterations such as the RB1 and TP53 mutations [18, 19, 21, 22]. RB1 and TP53 mutations have not been identified in well differentiated NETs. These genomic differences between well and poorly differentiated NETs suggest that the two subtypes of NETs are not the same disease. However, current treatment strategies do not reflect such clinicopathologic and genomic heterogeneities.

Personalized medicine means the use of an each patient’s molecular and clinicopathological data to inform diagnosis, prognosis, treatment, and prevention of cancer, and has become a primary goal of many studies in oncology [23]. The detection of actionabl genomic alterations has changed the paradaigm of cancer therapy into precision medicine in accordance with the development of genomic techniques [8, 9]. Hence, to improve our understanding of the heterogeneity of disease at the genomic and clinicopathologic levels is needed. Further, in heterogeneous GEP-NETs, studies are needed to understand biologic behavior and guide systemic therapy according to biologic characteristics.

The present study has some drawbacks, such as a too small sample size and heterogeneous patient population. There was also an inherent selection bias as retrospective natures, since only those G3 NE neoplasms receiving platinum were included. This means only the most aggressive of the well differentiated G3 NE tumors may have been included, since less aggressive G3 well differentiated NE tumors may not have been treated with platinum. Nevertheless, this analysis demonstrates that grade 3 GEP-NECs could be morphologically classified into well and poorly differentiated NETs. Additionally, among grade 3 GEP-NECs, there was a significant difference in ranges of Ki67 index between well and poorly differentiated NETs. Higher levels (> 60%) of Ki67 index might be a predictive marker for efficacy of EP as a standard regimen in grade 3 GEP-NECs.

## MATERIALS AND METHODS

### Patients

Among patients pathologically diagnosed with metastatic grade 3 GEP-NECs at Samsung Medical Center between June 2013 and March 2016, 31 GEP-NEC patients receiving etoposide and platinum-based therapy were included in this study. The following clinicopathologic characteristics were collected for all 31 patients: age, gender, primary site, pathologic differentiation, Ki67 index and information on chemotherapy. Tumors of all patients were reviewed and classified by grade according to the 2010 WHO classification using mitosis and Ki67 labeling index. Mitosis was reported as G1 (< 2/10 HPF), G2 (2–20/10 HPF), and G3 (> 20/10 HPF). The Ki67 labeling index was G1 (≤ 2%), G2 (3–20%), and G3 (> 20%)[4]. Based on embryological origin, tumors were classified as foregut (esophagus, stomach, duodenum, pancreas), midgut (appendix, ileum, cecum, ascending colon) and hindgut (distal large bowel, rectum) tumors [1]. Well differentiated categories comprise neoplastic cells uniform for size and features organized in organoid, trabecular, ribbon or gyriform architecture. They present abundant content of secretory granules responsible for intense and diffuse staining for general neuroendocrine markers (Synaptophysin and Chromogranins). Nuclear chromatin is regular with inconspicuous nucleoli, with no atypia. Mitoses are rare or uncommon. Poorly differentiated categories comprising large cell and small cell, are neoplasms with pleomorphic and highly atypical nuclei, solid growth pattern and abundant non-ischemic necrosis, arranged to form either map or spot necrosis. Mitoses are plentiful and often atypical.

### Statistical analyses

Descriptive statistics are reported as proportions and medians. Treatment outcomes were response rate (RR) and progression-free survival (PFS). Correlation between pathologic differentiation or Ki67 index and treatment outcome was analyzed using the *t*-test or Fisher’s exact test or by one-way analysis of variance, as appropriate. The best cut-off value for Ki67 regarding the response rate was determined from receiver operating characteristic (ROC) curve analysis. Progression free survival (PFS) was measured as the time from date of chemotherapy to the date of first documented disease progression or death. PFS was estimated using the Kaplan-Meier method with log-rank analysis. A two-sided *P*-value of less than 0.05 was considered significant. All analyses were performed using SPSS version 19.0 (SPSS Inc., Chicago, IL, USA).

## SUPPLEMENTARY MATERIALS TABLE


