# Supplementary material for: The impact of pathologic differentiation (well/poorly) and the degree of Ki-67 index in patients with metastatic WHO grade 3 GEP-NECs
Source: Oncotarget. 2017 May 25;8(43):73974–80. doi: 10.18632/oncotarget.18168 (PMC5650316; doi:10.18632/oncotarget.18168)
Supplement: Supplementary file 1 [file oncotarget-08-73974-s001.pdf]

The impact of pathologic differentiation (well/poorly) and the degree of Ki-67 index in patients with metastatic WHO grade 3 GEP-NECs

SUPPLEMENTARY MATERIALS

Supplementary Table 1: Details for 31 cases

Case	Ki67 (%)	Pathologic differentiation	Primary site
1	70%	Poorly	Gall bladder
2	78%	Poorly	Unknown primary origin
3	30%	Poorly	Liver
4	60%	Poorly	Gall bladder
5	40%	Well	Pancreas
6	28%	Well	Duodenum
7	40%	Poorly	Stomach
8	30%	Well	Pancreas
9	30%	Well	Liver
10	65%	Poorly	Duodenum
11	80%	Poorly	Gall bladder
12	40%	Well	Unknown primary origin
13	30%	Well	Liver
14	40%	Poorly	Stomach
15	50%	Poorly	Gall bladder
16	70%	Poorly	Gall bladder
17	45%	Poorly	Liver
18	38%	Poorly	Gall bladder
19	40%	Poorly	Unknown primary origin
20	30%	Well	Pancreas
21	42%	Poorly	Rectum
22	40%	Well	Stomach
23	40%	Well	Stomach
24	85%	Poorly	Rectum
25	30%	Well	Pancreas
26	60%	Well	Liver
27	30%	Well	Duodenum
28	60%	Poorly	Gall bladder
29	70%	Poorly	Duodenum
30	30%	Well	Pancreas
31	30%	Well	Liver
